# Pelvic floor parameters predict postpartum stress urinary incontinence: a prospective MRI study

**DOI:** 10.1186/s13244-023-01488-5

**Published:** 2023-09-27

**Authors:** Cong You, Yujiao Zhao, Cheng Zhang, Mengyao Chen, Wen Shen

**Affiliations:** 1 The First Central Clinical School, Tianjin Medical University, Nankai District, Tianjin, China; 2 Department of Radiology, Tianjin First Central Hospital, School of Medicine, Nankai University, No.24 Fukang Road, Nankai District, Tianjin, 300192 China

**Keywords:** Vaginal delivery, Stress urinary incontinence, Magnetic resonance imaging, Pelvic floor dysfunction, Pelvic floor

## Abstract

**Objective:**

To investigate the pelvic floor changes in primiparas with postpartum stress urinary incontinence (SUI) after vaginal delivery using pelvic floor MRI.

**Materials and methods:**

Fifty-two women were enrolled in the primiparous stress urinary incontinent (PSUI) group and 51 in the primiparous continent (PC) group. Thirty nulliparas were also recruited as the nulliparous control (NC) group. Levator ani muscle (LAM) injury, levator hiatus area (LHA), H-line, M-line, the distance from the bladder neck and cervix to the pubococcygeal line (B-PCL and U-PCL), levator plate angle, the anterior angle of the urethra, bladder neck descent, retrovesicourethral angle, functional urethral length, and a bladder neck funnel were evaluated on MRI images. Univariate and multivariate logistic regression analyses were used to explore anatomical predictors for SUI.

**Results:**

The primiparas in the PSUI group showed more obvious LAM injuries than in the PC groups (*p* = 0.001). LAM function assessment: the PSUI group had larger LHA and shorter B-PCL and U-PCL than the other groups during straining. Assessment of urethral mobility and function: the PSUI group had larger anterior angle of the urethra, bladder neck descent, retrovesicourethral angle, and shorter functional urethral length than the other two groups (all *p* < 0.05). Up to 88.5% of primiparas in the PSUI group showed bladder funnel (*p* < 0.001). The logistic regression analysis showed that retrovesicourethral angle, functional urethral length, and the presence of bladder funnel were significantly associated with postpartum SUI (*p* < 0.05).

**Conclusions:**

Increased retrovesicourethral angle, shortened functional urethral length, and the presence of bladder funnel may be anatomical predictors for SUI in the early postpartum period. Urethral sphincter dysfunction plays an essential role in developing postpartum SUI.

**Critical relevance statement:**

This study used several measurements to reflect the anatomical structure and functional changes of the pelvic floor to identify the best anatomical predictors associated with postpartum stress urinary incontinence (SUI), aiming to provide new insights into treatment strategies for postpartum SUI.

**Key points:**

• Increased retrovesicourethral angle, shortened functional urethral length, and the presence of bladder funnel are more commonly seen in primiparas with SUI.

• The combination of retrovesicourethral angle, functional urethral length, and bladder funnel had the highest diagnostic performance in predicting postpartum SUI (AUC=0.947).

• Urethral sphincter dysfunction may be the main pathophysiological foundation in SUI development.

**Graphical Abstract:**

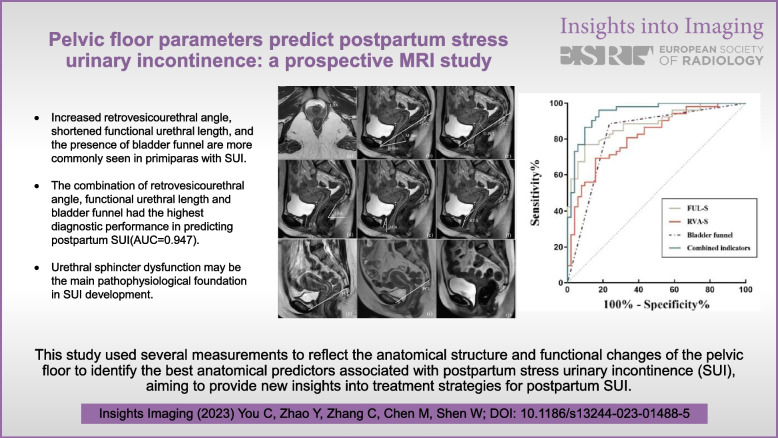

## Introduction

Stress urinary incontinence (SUI) manifests as urine leakage during certain physical activities such as coughing, sneezing, laughing, or exercising, increasing intra-abdominal pressure [1]. It is the most common type of urinary incontinence in women. Vaginal delivery is a known risk factor for SUI, especially after the first childbirth [2, 3]. About 26% of primiparas suffer from SUI after delivery, significantly affecting their physical health and quality of life [4]. The physiological changes during pregnancy and childbirth affect the pelvic floor muscles, nerves, and fascial tissues, essential to developing postpartum SUI.

It has been reported that about 29% of SUI surgeries were repeat procedures [5], indicating that a timely clinical intervention targeting key pelvic floor abnormalities is crucial for treating SUI. Our previous studies have shown that LAM injury, urethral hypermobility, and urethral sphincter dysfunction were responsible for postpartum SUI [6]. However, the small sample size is insufficient to definite the effect of specific antonymic abnormalities in the development of postpartum SUI. Moreover, few studies attempted to seek the best predictive parameters of postpartum SUI, covering all pelvic floor parameters related to LAM, urethral mobility, and urethral sphincter on MRI images.

Therefore, our study aimed to comprehensively assess the pelvic floor structural changes in primiparas with SUI in the early postpartum period using pelvic floor MRI and to seek the best anatomical predictors associated with postpartum SUI. We hope to provide new insights into postpartum SUI and treatment strategies in this study.

## Materials and methods

### Subjects

**Fig. 1:**
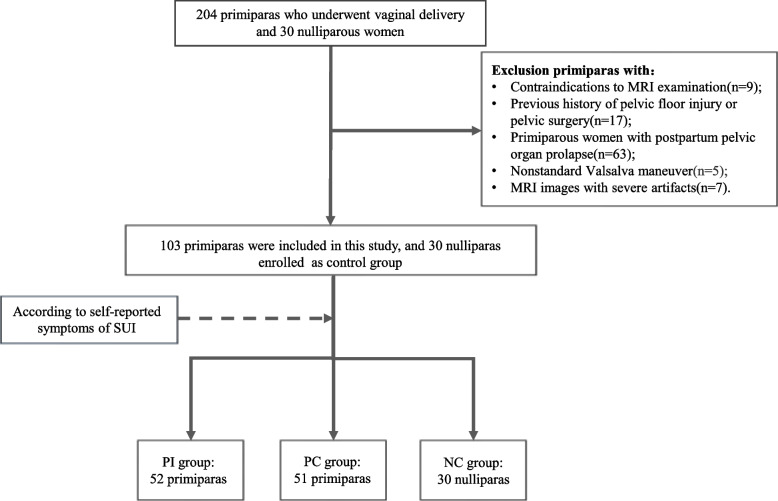
Flowchart of patient selection. PSUI group, primiparous stress urinary incontinent group; PC group, primiparous continent group

The study was approved by the clinical research ethics committee of our hospital. Primiparas at 6 weeks postpartum after vaginal delivery in our hospital were prospectively recruited from October 2018 to December 2020. All the subjects signed informed consent before enrollment and underwent a pelvic floor MRI. The inclusion criteria were as follows: (1) primipara, with natural conception; (2) singleton pregnancy; (3) term delivery, with gestational age of more than 37 weeks. The exclusion criteria were (1) previous history of pelvic floor injury, pelvic surgery, or pelvic floor dysfunction, (2) primiparas with postpartum pelvic organ prolapse, (3) primiparas with contraindications for MRI examination, (4) MRI images with severe artifacts, and (5) invalid Valsalva maneuver performed during the examination. A total of 103 women were enrolled in our study. The International Continence Society (ICS) defines SUI as the involuntary passage of urine during a sudden increase in intra-abdominal pressure (coughing, sneezing, laughing, or exercise). The primiparas were stratified based on the presence of the above symptoms of SUI. Finally, 52 women were enrolled in the primiparous stress urinary incontinent (PSUI) group and 51 in the primiparous continent (PC) group. 30 nulliparas were also recruited as the nulliparous control (NC) group, and the inclusion criteria for nulliparous women were: no abnormalities on clinical examination and imaging examination performed. The flowchart of study recruitment is presented in Fig. 1.

### MRI imaging

#### Preparation

All participants were asked to empty their bladder 1 h before the examination and then drink 300 mL of water, to maintain optimal bladder filling before the examination. No gel was used in the vagina and rectum in this study. One physician trained all the primiparas to perform the Valsalva maneuver before MRI until they understood the essential steps of the maneuver. All the participants were placed in a supine position with a soft wedge-shaped pad under their knees to simulate a lithotomy position to assist force and asked to breathe calmly during the MRI examination.

#### MRI protocols

All the participants underwent pelvic floor MRI performed with a 3.0-T unit (Ingenia, Philips Healthcare) and a 16-channel body coil. The pelvic floor MRI examination protocol included a multi-planar localizer, T2 weighted fast spin echo (FSE) sequence, T2 weighted balanced fast field echo (balanced FFE), and single-shot fast spin-echo sequence. Axial, sagittal, and coronal T2 fast spin echo (FSE) were obtained rest (3 mm/0.3 mm gap; field of view 28 cm; TR/TE 4110/102 ms). The subjects were then asked to perform the Valsalva maneuver during the balanced FFE examination (5 mm/0.5 mm gap; field of view 30 cm; TR/TE 4818/95 ms; turn angle 90°). The action was repeated 2–3 times to obtain optimal images. Axial, sagittal, and coronal single-shot fast spin-echo images were obtained during the maximum Valsalva maneuver (6 mm/0.6 mm gap; field of view 28 cm; TR/TE 4.8/2.4 ms). The total time for pelvic floor examination time varied from 15 to 20 min.

#### Imaging analysis

Imaging analyses and measurements were performed on the Picture Archiving and Communication System (PACS) workstation. Two radiologists (with more than five years of experience in diagnostic genitourinary imaging) reviewed the images by consensus. All the measurements were taken at rest and straining and are presented in Table 1.

**Table 1:** Morphological measurements

	Measurements	Description
Axial plane	LAM scoring	The LAM scoring system was used to assess the presence of LAM injury at the level of the inferior edge of the pubic symphysis
	LHA	The area enclosed by the medial border of bilateral puborectalis muscle at the level of the inferior border of the pubic symphysis
Sagittal plane	H line	Distance from the inferior margin of the pubic symphysis to the posterior rectal wall at the anorectal junction
	M line	Distance from the most distal H-line to the vertical line on the PCL
	B-PCL	The vertical distance from the bladder neck to the PCL represents the position of the bladder neck, which is marked with a positive sign above PCL and a negative sign below
	U-PCL	The vertical distance from the cervix to PCL represents the position of the cervix.
	LPA	The angle between the levator plate and horizontal line
	AUA	The angle between the long axis of the urethra and the vertical line
	BND	The difference in distance from the bladder neck to PCL line at rest and straining
	RVA	The angle between the long axis of the urethra and the posterior wall of the bladder
	FUL	Distance from the lowest point of the bladder neck to the urogenital diaphragm
	Bladder funnel	The widening of the proximal urethra at the ureterovesical junction and funneling change at the vesical neck during straining, the presence-absence or of bladder funnel was recorded as positive or negative

*LPA* Levator plate angle, *AUA* Anterior angle of the urethra, *BND* Bladder neck descent, *RVA* retrovesicourethral angle, *FUL* Functional urethral length

#### Pelvic floor measurements

**Fig. 2:**
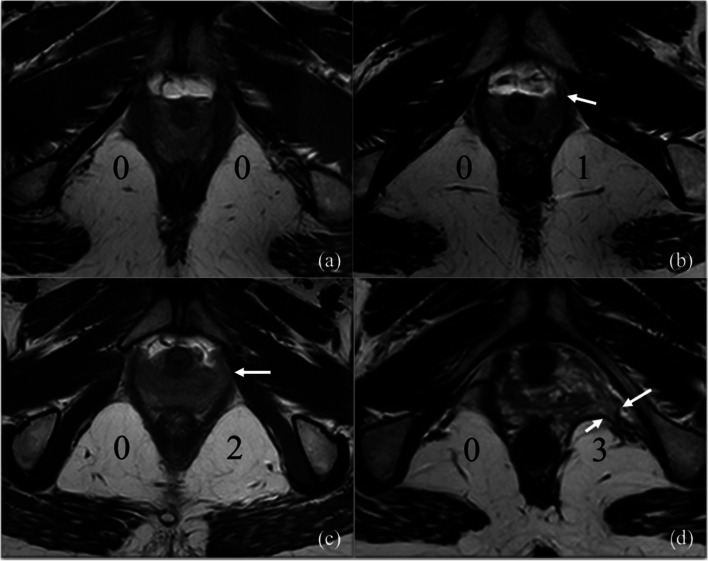
Axial T2-weighted images were performed to assess levator ani injury. The diagnosis of abnormal muscle was based on muscle swelling and defect. **a** 0 = normal, **b** 1 = less than half abnormality of the muscle, **c** 2 = more than half abnormality of the muscle, **d** 3 = total or near-total loss of the muscle

The LAM injury score: the muscle injury was assessed according to the LAM scoring system proposed by DeLancey et al. [7]. The muscles on both sides were separately scored as follows: score “0” (normal), “1” (if less than half of the muscle was injured), “2”(if more than half of the muscle was injured but not completely disrupted), and “3” (if the muscle was disrupted entirely). The total scores, including two sides, ranged from 0 to 6: 0 indicated no defect, 1–3 indicated a minor defect, and 4–6 indicated a major defect. A unilateral score of 3 indicated a major defect (Fig. 2).

**Fig. 3:**
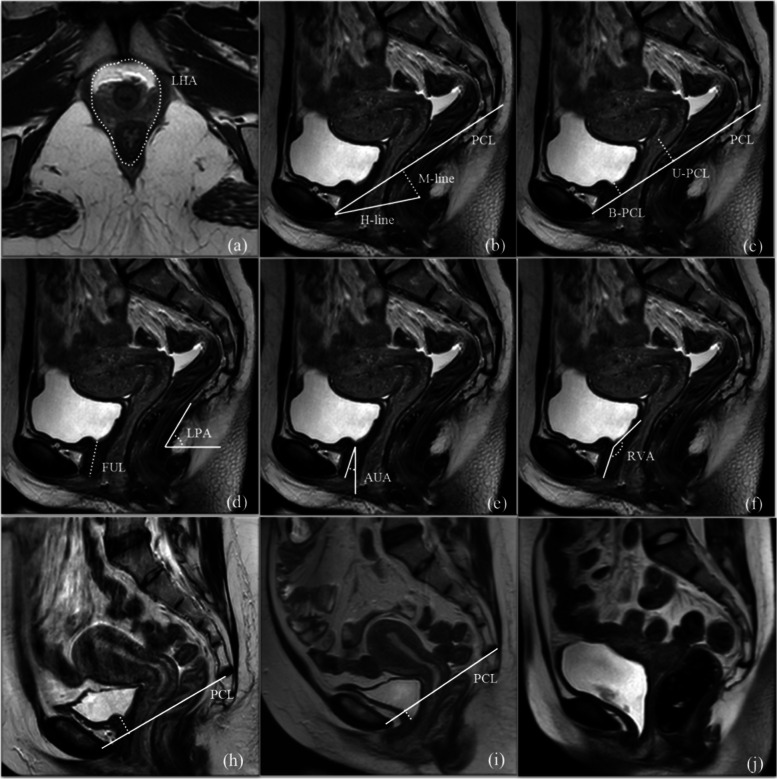
Schematic diagram of pelvic floor measurements. **a** Axial T2-weighted images. LHA, levator hiatus area. **b**–**f** Median sagittal T2-weighted images. H line, puborectal hiatus line; M line, muscular pelvic floor relaxation line; B-PCL, bladder-pubococcygeal line; U-PCL, uterus-pubococcygeal line; FUL, functional urethral length; LPA, levator plate angle; AUA, anterior angle of the urethra; RVA, retrovesicourethral angle; all the parameters were measured at rest and straining respectively; **h**, **i** bladder neck descent is defined as the difference in distance from the bladder neck to the PCL line at rest and straining. Negative value was taken if the bladder neck was located below PCL. **j**, incomplete bladder neck closure in a primiparous incontinent woman manifested as bladder neck funnel (arrows)

Functions of the levator ani muscle: LHA indicates the ability of the levator ani muscle to close the levator hiatus area; H-line indicates the ability of the levator ani muscle to tighten three pelvic organs, namely, the urethra, uterus, and rectum longitudinally; M-line, reflects the degree of cephalocaudal movement of the pelvic organs; B-PCL and U-PCL, indicate the position of the bladder and uterus; levator plate angle, reflects the ability of the levator ani plate to tighten the posterior pelvic organs (Fig. 3).

Mobility of the urethra: the anterior angle of the urethra reflects urethral mobility, and bladder neck descent reflects bladder neck mobility (Fig. 3).

Assessment of the urethral sphincter function: retrovesicourethral angle [8] indicates the integrity of closure of the bladder neck; functional urethral length indicates the urethral sphincter function (Fig. 3); Bladder funnel indicates the ability of closure of the internal urethral meatus and also indirectly reflects the expansion ability of the urethral sphincter (Fig. 3).

### Statistical analysis

Statistical analyses were performed using the SPSS version 26.0 (IBM, Armonk, NY). *p* < 0.05 was considered statistically significant. The intraclass correlation coefficient (ICC) was used to evaluate the interobserver variation of all the measurements.

The variables are presented as absolute numbers, mean ± standard deviation, or absolute numbers and percentages. The normality of the distribution was tested using the Kolmogorov-Smirnov test. Comparisons between the three groups were made using the Chi-square test for categorical variables and one-way analysis of variance or the Wilcoxon rank-sum test for quantitative variables. The Bonferroni method was used for the post hoc analysis. Demographic data of the primipara were compared between the PSUI and PC groups using the chi-square test for categorical variables and independent-sample *t*-tests or the Mann-Whitney *U* test for quantitative variables.

Pelvic floor variables independently associated with SUI in univariate analysis were considered for the multivariate logistic regression model. All models were fitted using a stepwise forward method with a removal probability of 0.1. The performance of significant variables in logistic regression analysis to predict SUI was further assessed by calculating the area under the receiver operating characteristic (ROC) curve.

## Results

### Study population

The characteristics of the study population are shown in Table 2. The primiparas’ age in the PSUI group was higher than that in the PC and NC groups, and the BMI was also higher than that in the PC and NC groups (*p* < 0.001); however, there was no difference between the PC group and the NC group. There were significant differences in the fetal weight and duration of the second stage of labor between the PSUI and PC groups (all *p* < 0.05); however, there were no significant differences in other obstetric characteristics between the PSUI and PC groups (all* p *> 0.05).

**Table 2:** Demographic data

Characteristic	PSUI group (*N *= 52)	PC group (*N *= 51)	NC group (*N *= 30)	*p* value
Age (years)	30.7 ± 3.6^a,b^	27.7 ± 3.6	26.5 ± 2.8	< 0.001^*^
BMI (kg/m^2^)	24.7 ± 3.2^a,b^	23.0 ± 2.8	21.6 ± 1.5	< 0.001^*^
Fetal weight (g)	3348.8 ± 400.6	3141.8 ± 542.9	-	0.033^*^
Biparietal diameter (mm)	92.3 ± 2.8	90.8 ± 4.9	-	0.099
The second stage of labor (mins)	104.4 ± 16.7	85.4 ± 13.3	-	< 0.001^*^
Perineal tear (%)	21.2 (11/52)	17.6 (9/51)	-	0.804
Forceps-assisted delivery (%)	25.0 (13/52)	11.8 (6/51)	-	0.126

*BMI*, body mass index

^*^represents statistical significance

^a^represents statistical difference compared with the PC group, and

^b^represents statistical difference compared with the NC group

### Comparison of morphology and function of levator ani muscle between different groups

**Fig. 4:**
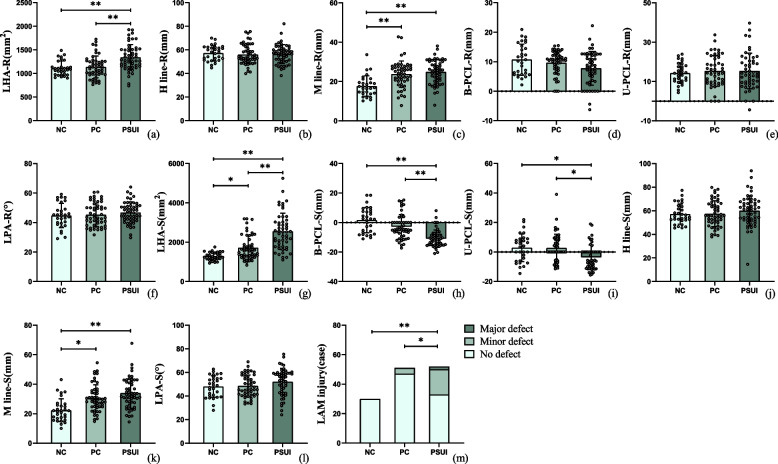
Prism plot for comparison of morphology and function of the levator ani muscle. **a**–**f** The comparison of each pelvic floor parameter at rest; **g**–**l** the comparison of each pelvic floor parameter during straining; **m** the constituent ratio of LAM injury between the three groups. LPA, levator plate angle; * represents *p* < 0.05, ** represents *p* < 0.001

During the assessment of the morphology of the levator ani muscle, 32.7% (17 cases) of women in the PSUI group had minor defects of the LAM, and 3.8% (2 cases) had major defects. In contrast, 7.8% (4 cases) of women in the PC group had minor defects. No defects in the LAM were observed in any of the nulliparous women. The results of Fisher’s exact statistics showed that there were statistical differences in the proportion of LAM injuries between the PSUI group and PC group (*p* < 0.001) and the PSUI group and NC group (*p = *0.005), suggesting that LAM injury was associated with postpartum SUI (Table 3, Fig. 4).

**Table 3:** Comparison of morphology and function of levator ani muscle

		PSUI group	PC group	NC group	*p* value
LAM injury (%)	None	63.5 (33/52)	92.2 (47/51)	100 (30/30)	< 0.001^*^
	Minor	32.7 (17/52)	7.8 (4/51)	0	
	Major	3.8 (2/52)	0	0	
LHA (mm^2^)	Rest	1339.9 ± 272.6^a,b^	1136.2 ± 231.8	1107.6 ± 156.1	< 0.001^*^
	Strain	2552.7 ± 924.6^a,b^	1719.7 ± 617.7^b^	1292.9 ± 204.2	< 0.001^*^
H-line (mm)	Rest	56.4 ± 8.0	56.1 ± 8.6	57.2 ± 6.5	0.824
	Strain	60.0 ± 13.0	57.5 ± 11.0	57.1 ± 8.6	0.413
M-line (mm)	Rest	24.9 ± 6.1^b^	23.8 ± 6.7^b^	17.7 ± 5.2	< 0.001^*^
	Strain	33.9 ± 10.1^b^	30.5 ± 9.1^b^	22.4 ± 7.7	< 0.001^*^
B-PCL (mm)	Rest	7.8 ± 5.7	9.7 ± 3.9	10.7 ± 5.3	0.105
	Strain	−11.0 ± 5.9^a,b^	−2.9 ± 8.2	1.6 ± 8.6	< 0.001^*^
U-PCL (mm)	Rest	15.3 ± 9.0	15.3 ± 7.8	14.3 ± 5.2	0.807
	Strain	−3.6 ± 8.2^a,b^	2.7 ± 9.4	1.6 ± 8.6	< 0.001^*^
LPA (°)	Rest	46.8 ± 7.2	45.3 ± 8.0	44.8 ± 8.2	0.453
	Strain	52.0 ± 11.3	48.5 ± 9.2	48.1 ± 9.1	0.089

*LPA* Levator plate angle

^*^represents statistical significance

^a^represents statistical difference compared with the PC group, and

^b^represents statistical difference compared with the NC group

The assessment of LAM function showed a larger LHA and shorter B-PCL and U-PCL in the PSUI group compared to that in the PC and NC groups during straining (all *p* < 0.05). The M line in the PSUI and PC groups was longer than that in the NC group, and the PC group had a larger LHA compared to the NC group (all *p* < 0.05). At rest, the PSUI group had a larger LHA compared to the other two groups, and the PSUI and PC groups had a longer M-line compared to the NC group (all *p* < 0.05) (Table 3, Fig. 4).

### Comparison of urethral mobility and urethral sphincter function between different groups

During the assessment of urethral mobility, the anterior angle of the urethra and bladder neck descent in the PSUI group were larger than that in the PC and NC groups, with the PC group having a larger anterior angle of the urethra than the NC group during straining (all *p* < 0.05). Moreover, the PSUI group also had a larger anterior angle of the urethra compared to the NC group at rest (*p = *0.006).

**Fig. 5:**
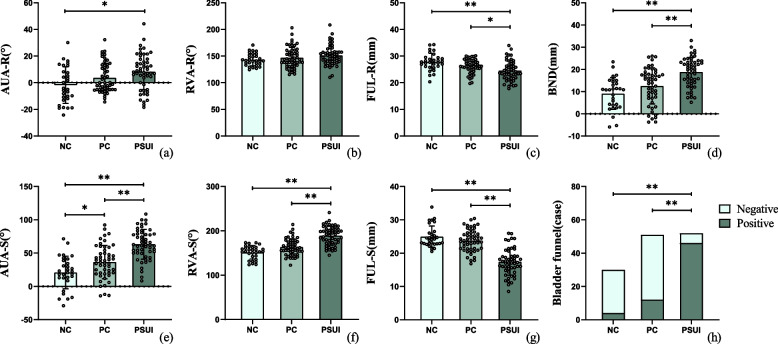
Prism plot for comparison of urethral mobility and urethral sphincter function. **a**–**c** The comparison of each pelvic floor parameter at rest; **e**–**g** the comparison of each pelvic floor parameter during straining; **d** the descent distance of the bladder neck during the Valsalva maneuver; **h** the constituent ratio of the bladder funnel between the three groups. AUA, anterior angle of the urethra; RVA, retrovesicourethral angle; FUL, functional urethral length; BND, bladder neck descent; * represents *p* < 0.05, ** represents *p* < 0.001

On analyzing the urethral sphincter function, the PSUI group had a larger retrovesicourethral angle compared to the PC and NC groups during straining (*p* < 0.001); however, there was no statistical difference in the retrovesicourethral angle between the three groups at rest (*p = *0.112). The functional urethral length at rest and straining was shorter in the PSUI group compared to the PC and NC groups (all *p* < 0.05). Bladder funnel was seen in all three groups, and the proportions were as follows: PSUI group (88.5%), PC group (23.5%), and nulliparous group (11.8%). The difference in the proportion of bladder funnels was statistically significant (*p* < 0.001) (Table 4, Fig. 5).

**Table 4:** Comparison of urethral mobility and urethral sphincter function

		PSUI group	PC group	NC group	*p* value
AUA (°)	Rest	8.2 ± 13.4^b^	3.6 ± 11.0	-1.8 ± 13.7	0.006^*^
	Strain	63.2 ± 22.3^a,b^	36.5 ± 25.0^b^	20.8 ± 24.5	< 0.001^*^
BND (mm)	-	18.8 ± 6.5^a,b^	12.5 ± 8.1	9.1 ± 7.2	< 0.001^*^
RVA (°)	Rest	151.1 ± 18.4	146.9 ± 19.5	143.3 ± 11.6	0.113
	Strain	188.9 ± 21.2^a,b^	163.2 ± 18.7	151.4 ± 15.0	< 0.001^*^
FUL (mm)	Rest	24.5 ± 3.7^a,b^	26.1 ± 2.6	27.8 ± 3.2	< 0.001^*^
	Strain	17.4 ± 4.0^a,b^	23.7 ± 3.3	24.9 ± 3.2	< 0.001^*^
Bladder funnel (%)	Strain	88.5 (46/52)	23.5 (12/51)	13.3 (4/30)	< 0.001^*^

*AUA* Anterior angle of the urethra, *BND* Bladder neck descent, *RVA* Retrovesicourethral angle, *FUL* Functional urethral length

^*^represents statistical significance

^a^represents statistical difference compared with the PC group, and

^b^represents statistical difference compared with the NC group

### Independent anatomical predictors of stress urinary incontinence

The results of logistic regression analysis with the forward method showed that the retrovesicourethral angle during straining(retrovesicourethral angle-S) [OR 1.038, 95% CI (1.007~1.069)], functional urethral length during straining (functional urethral length-S) [OR 0.706, 95% CI (0.580, 0.859)], and the presence of bladder funnel during straining [OR 10.360, 95% CI (2.726, 39.372)] were predictors of postpartum SUI (Table 5).

**Table 5:** Multivariate analysis and ROC curve of imaging predictors for SUI

Parameters	Logistic regression analysis			ROC analysis			
	OR	95% CI	*p* value	AUC	Threshold	Sensitivity (%)	Specificity (%)
FUL-S (mm)	0.706	(0.580, 0.859)	0.001^*^	0.882	19.4	76.9%	90.2%
RVA-S (°)	1.038	(1.007, 1.069)	0.015^*^	0.816	179.2	69.2%	84.3%
Bladder funnel	10.360	(2.726, 39.372)	0.001^*^	0.825	-	88.5%	76.5%
Combined indicators	-	-	-	0.947	-	96.2%	82.4%

*OR* Odds ratio, *95% CI* 95% confidence interval, *AUC* Area under the curve, *FUL-S* Functional urethral length, *RVA-S* Retrovesicourethral angle; both were measured during straining

^*^ represents statistical significance

### Diagnostic performance of anatomical predictors

**Fig. 6:**
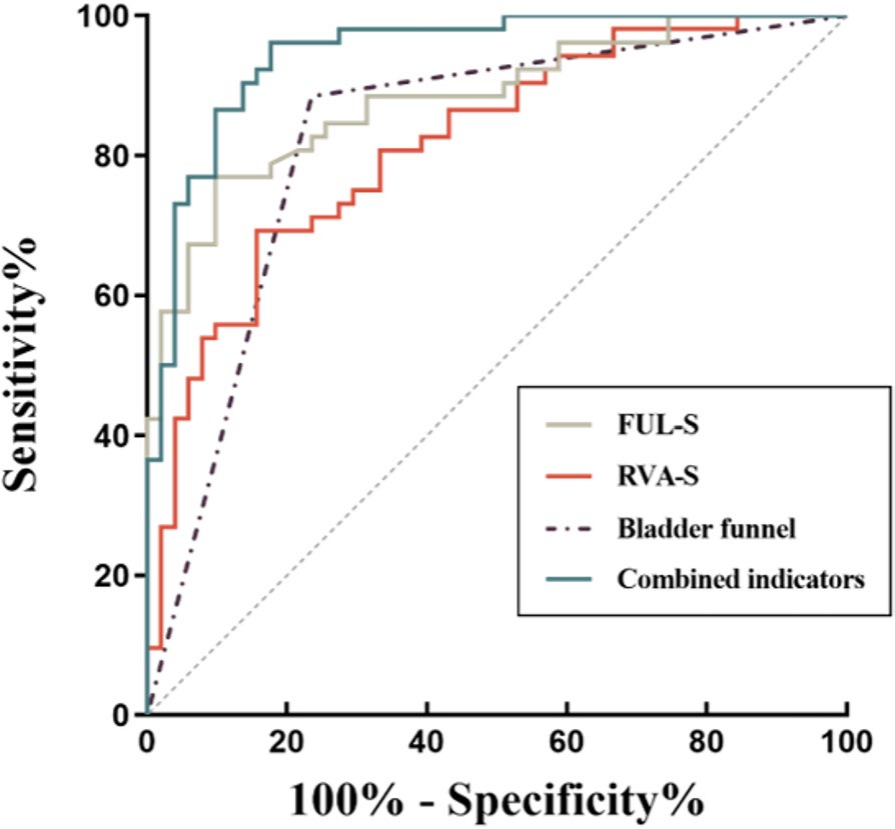
ROC curve of pelvic floor parameters for predicting postpartum SUI. FUL-S, functional urethral length; RVA-S, retrovesicourethral angle; both were measured during straining

The performances of retrovesicourethral angle-S, functional urethral length-S, and bladder funnel in predicting postpartum SUI are shown in Table 5 and Fig. 6. In the ROC analysis, the AUCs for predicting postpartum SUI was 0.816 using retrovesicourethral angle-S, 0.882 using functional urethral length-S, and 0.825 using bladder funnel. When the retrovesicourethral angle-S, functional urethral length-S, and bladder funnel were combined, the AUC significantly improved to 0.947 for predicting postpartum SUI, with a corresponding sensitivity of 96.2% and specificity of 82.4%.

## Discussion

Our study found that the PSUI group had higher age, higher BMI, and higher fetal weight than the other groups, possibly because the transverse urethral sphincter was reduced with age. Previous studies have demonstrated that high fetal weight and BMI can increase bladder pressure and might impair the blood supply and innervation to the bladder and urethra, thus, leading to continence disorder [9]. We also found that the second stage of labor was significantly prolonged in the PSUI group. The fetal head compresses the nerve tissue and the pelvic floor muscles for a long time during an abnormal second stage of labor, resulting in pelvic floor muscle fatigue and damage to its function [10].

This study used a series of pelvic floor measurements to reflect structural and functional changes in the postpartum pelvic floor. An important point is that the effect of bladder fullness on certain pelvic floor measurements is unknown. However, In this study, the subjects were already asked to empty their bladder and drink 300ml of water 1 h before the scan to maintain a moderate and uniform bladder fullness state, so as to eliminate the influence of bladder fullness degree on pelvic floor measurement as far as possible. As reported by Delancey et al. [7], we found that the primiparas had different degrees of LAM injury while the nulliparous women had no injuries, suggesting that vaginal delivery was responsible for LAM injury. Moreover, the PSUI group had minor and major LAM injuries, while the PC group mainly had a small percentage of minor injuries, indicating an inseparable relationship between the decreased function of the LAM and subsequent postpartum SUI [11].

Moreover, there were significant differences in the pelvic floor parameters related to LAM function at rest and straining. The PSUI group had a larger LHA and shorter B-PCL and U-PCL. Meanwhile, the PSUI and PC groups had longer M lines than the NC group, and the PC group had larger LHA than the NC group. These changes were attributed to hormonal changes during pregnancy and extreme stretching or denervation of the LAM during vaginal delivery, which might lead to pelvic floor relaxation and reduce the support to the pelvic organs, eventually resulting in overactive movement of pelvic organs [12, 13].

Compared to the PC and NC groups, the PSUI group had larger AUA and BND during straining, indicating that the urethral mobility of the PSUI group increased significantly. The anterior vaginal wall, periurethral ligaments, and LAM provide hammer-like support to the bladder neck and the urethra. Injury to the periurethral support structures during vaginal delivery results in unstable urethral support and adverse anatomical position, manifesting as urethral hypermobility and rotational downward movement [14, 15]. These anatomical abnormalities are inadequate to maintain a normal pressure conduction system, leading to urine leakage [16, 17].

The integrity of bladder neck closure was deficient in the PSUI group, as indicated by increased retrovesicourethral angle during straining, possibly due to decreased external support for the urethra and bladder neck [18]. Functional urethral length refers to the length of the urethra in which the urethral pressure is higher than the intravesical pressure. Previous studies have reported that SUI can be attributed to a shorter urethra or a smaller volume of the urethral muscle [19]. Our study confirms that primiparas with SUI had a shorter functional urethral length. The bladder funnel has been associated with lower maximum urethral closure pressure, reflecting the decrease in urethral sphincter function [20]. In our study, the proportion of bladder funnel in primiparas with SUI was up to 88.5%, indicating a decreased tension of the urinary sphincter and reduced function of maintaining urethral closure in women in the PSUI group.

Based on a previous study [6], we explored the main pathophysiological factors and anatomical predictors of SUI in the early postpartum period. We included primiparous women 42 days after delivery instead of 6 months after delivery in this study. However, the multivariate logistic regression showed that only increased retrovesicourethral angle, shortened functional urethral length, and the formation of bladder funnel during straining were risk factors for postpartum SUI. The functional urethral length is related to the maximum urethral closure pressure. After vaginal delivery, the pelvic floor structure is in a relaxed state. The downward displacement of the bladder neck results in shortened functional urethral length, resulting in the decrease of maximum urethral closure pressure. When the abdominal pressure increases, the urethral pressure cannot be effectively increased but is lower than the increased intravesical pressure, thus promoting the occurrence of SUI [21]. The retrovesicourethral angle and the bladder funnel both reflect the abnormal state of the bladder neck, the increased retrovesicourethral angle suggests a lack of closure integrity at the vesicourethral junction, while it means the appearance of the bladder funnel [22]. All of these significantly suggest that there are functional defects in the urethral sphincter, which further reduces the function of maintaining the urethral closure and decreases continence. However, the weak correlation between LAM injury, urethral hypermobility, and SUI indirectly suggest that urethral sphincter dysfunction might be the main pathogenic factor for SUI. Hormonal regulation during pregnancy causes increased degradation of collagen fibers of the urethral sphincter and pudendal nerve injury during vaginal delivery, causing denervation of the urethral sphincter leading to urethral sphincter dysfunction. This is manifested as reduced urethral sphincter tension caused by incomplete closure of the internal urethral meatus prone to leakage [23]. These findings highlight the importance of urethral sphincter function in the continence mechanism.

ROC curve analysis showed that retrovesicourethral angle, functional urethral length, and bladder funnel formation during straining had a high predictive ability for postpartum SUI (all AUC > 0.8). Functional urethral length-S had the largest AUC, with high specificity (90.2%) and relatively low sensitivity of 76.9%. The AUC of the comprehensive index obtained from the logistic regression analysis was 0.947, with high sensitivity (96.2%) and specificity (82.4%). The combination of multiple risk factors can further improve the predictive performance, providing an objective imaging reference for disease monitoring and treatment options or evaluation.

Our study has several limitations. First, the limited sample size and numerous pelvic floor parameters might have affected the accuracy of multivariate logistic regression. Second, this study did not investigate the relationship between the pelvic floor parameters and the severity of SUI. A further study focusing on the correlation between parameters and severity using the international consultation on incontinence questionnaire-short form (ICIQ-SF) might be necessary. Third, due to the lack of corresponding follow-up data, it is still unclear whether the pelvic floor parameters can predict the recovery or progression of postpartum SUI.

In conclusion, urethral sphincter dysfunction might be the leading pathophysiological foundation for SUI. The increased retrovesicourethral angle, shortened functional urethral length, and the presence of a bladder funnel can be used to predict and monitor postpartum SUI.

## Data Availability

Data sharing is not applicable to this article as no datasets were generated or analyzed during the current study.

## Abbreviations

AUC: Area under the curve
FFE: Fast field echo
FSE: Fast spin echo
ICC: Intraclass correlation coefficient
ICIQ-SF: International consultation on incontinence questionnaire-short form
ICS: International Continence Society
LAM: Levator ani muscle
LHA: Levator hiatus area
MRI: Magnetic resonance imaging
PACS: Picture Archiving and Communication System
PCL: Pubococcygeal line
ROC: Receiver operating characteristic
SUI: Stress urinary incontinence
